# Exploring the Pleiotropic Cardioprotective Effects of GLP-1 Receptor Agonists in Preventing Anthracycline-Induced Cardiotoxicity: A Theoretical Proposal for Future Research

**DOI:** 10.3390/medicines13010010

**Published:** 2026-03-17

**Authors:** Matthew L. Repp, Ikeotunye Royal Chinyere, Santiago Teran, Julia Bast, Lavanya Kondapalli

**Affiliations:** 1 Division of Internal Medicine, Department of Medicine, University of Colorado Anschutz Medical Campus, Aurora, CO 80045, USA; santiago.teran@cuanschutz.edu (S.T.); julia.bast@cuanschutz.edu (J.B.); 2 Sarver Heart Center, University of Arizona, 1501 North Campbell Avenue, Room 6154, Tucson, AZ 85724, USA; ichinyere@arizona.edu; 3 Division of Cardiology, Department of Medicine, University of Colorado Anschutz Medical Campus, Aurora, CO 80045, USA; lavanya.kondapalli@cuanschutz.edu

**Keywords:** anthracyclines, cardiotoxicity, cardio-oncology, glucagon-like peptide 1 receptor agonists, heart failure

## Abstract

Glucagon-like peptide-1 receptor agonists (GLP-1 RAs) have been shown to reduce morbidity and mortality associated with type II diabetes mellitus, and/or obesity, and/or cardiovascular disease in multiple clinical trials. Their efficacy in reversing cardiovascular disease and mitigating the risk of major adverse cardiac and vascular events has been well studied, with outcome trials consistently demonstrating benefits such as reduced systemic inflammation, improved endothelial function, and favorable metabolic effects. These pleiotropic actions have nearly innumerable potential applications, with a progressively growing interest in using GLP-1 RAs to mitigate increased cardiovascular disease risk secondary to other off-target pharmacologic agents. Given these effects, the potential to utilize GLP-1 RAs for prophylactic cardioprotection before, during, and/or after chemotherapy regimens is of great interest. These effects are thought to be mediated in part through anti-inflammatory and antioxidant mechanisms that counter inflammation and reactive oxygen species-driven myocardial injury central to anthracycline-induced cardiotoxicity (AIC). Anthracyclines, a widely used class of chemotherapeutics for various malignancies, are frequently associated with dose-dependent and often irreversible cardiotoxicity, leading to heart failure, reduced quality of life, and adverse long-term outcomes. For the past three decades, dexrazoxane has been the sole Food and Drug Administration-approved agent for cardioprotection in this setting. However, in the current era of novel therapies with multi-system benefits—such as GLP-1 RAs—we propose a theoretical framework exploring their potential role in mitigating AIC and underscore the need for further clinical investigation in this new arena in the field of cardio-oncology.

## 1. Introduction

Anthracyclines were first discovered in the 1960s as antibiotic compounds derived from *Streptomyces peucetius* and *Streptomyces caesius*, and were later found to possess potent antitumor activity [1]. Following their discovery, anthracyclines underwent extensive clinical investigation, ultimately leading to the discovery of their chemotherapeutic effects in clinical oncology, with doxorubicin receiving its first Food and Drug Administration (FDA) approval in 1974 for the treatment of various malignancies [2]. Anthracyclines have remained a cornerstone in the treatment of breast cancer, lymphomas, acute leukemias, and soft tissue sarcomas, and continue to be utilized in other hematologic and solid malignancies [3]. Early in its utilization, anthracycline-induced cardiotoxicity (AIC) became a well-documented adverse effect manifesting in clinical heart failure [4,5], with a nearly 50% mortality rate for those with high cumulative exposures [6]. The dose-dependent risk of cardiotoxicity for doxorubicin increases beyond 400–450 mg/m^2^, with estimated risks of 1–5% up to 550 mg/m^2^, 30% at 600 mg/m^2^, and 50% at 1000 mg/m^2^ or higher. For epirubicin, cardiotoxicity is generally observed at cumulative doses above 900 mg/m^2^ [7,8]. Despite ongoing research, AIC remains a significant clinical challenge, largely due to the limited availability of effective prophylactic agents and/or strategies.

Over the past several decades, numerous cardioprotective agents have been investigated in randomized controlled trials for the prevention and treatment of anthracycline-induced heart failure. These agents include N-acetylcysteine, phenethylamines, coenzyme Q10, a combination of vitamin E, vitamin C, and N-acetylcysteine, L-carnitine, carvedilol, and amifostine—none of which have demonstrated a significant benefit in preventing AIC and subsequent heart failure [9]. Dexrazoxane is the only FDA-approved cardioprotective agent for the prevention of both clinical and subclinical anthracycline-induced heart failure. Nearly a dozen clinical trials have demonstrated that patients receiving dexrazoxane with anthracycline treatment have approximately one-third the risk of developing AIC compared to those receiving anthracyclines alone, with no impact on chemotherapy response rate or survival [9]. Unfortunately, dexrazoxane is not suitable for all cancer patients. In the United States, dexrazoxane is approved for women with metastatic breast cancer who have received high (≥300 mg/m^2^) doses of doxorubicin and require continued treatment. There remains a critical need for novel cardioprotective therapies for the diverse patients undergoing anthracycline-based treatment regimens. Despite decades of additional research into preventative strategies, dexrazoxane remains the only FDA-approved therapy for AIC. However, its limited indications underscore the pressing need for alternative strategies, particularly ones that leverage our growing understanding of inflammation and oxidative stress as central drivers of AIC.

There is growing interest, supported by early evidence, in the use of cardiometabolic agents as cardioprotective therapies for patients undergoing high-dose anthracycline treatment. In particular, antidiabetic medications such as sodium–glucose cotransporter 2 inhibitors (SGLT2is) and glucagon-like peptide-1 receptor agonists (GLP-1 RAs) have attracted considerable attention for their potential to mitigate AIC. SGLT2is have demonstrated efficacy in both heart failure with reduced and preserved ejection fraction and have been associated with a reduction in the incidence of anthracycline-induced cardiac dysfunction [10]. The EMPACT trial (Empagliflozin in the Prevention of Cardiotoxicity in Cancer Patients Undergoing Chemotherapy Based on Anthracyclines; NCT05271162) is currently investigating this therapeutic potential. Randomized, double-blind, placebo-controlled trials have demonstrated that the GLP-1 RAs semaglutide and liraglutide reduce the incidence of cardiovascular death and nonfatal myocardial infarction in patients with type II diabetes mellitus (T2DM) at high cardiovascular risk [11,12]. GLP-1 RAs exert pleiotropic cardiovascular effects, including anti-inflammatory, antioxidant, and anti-apoptotic actions that directly target the pathophysiological mechanisms underlying anthracycline-induced myocardial injury. The pleiotropic effects of GLP-1 RAs, demonstrated in both preclinical models and clinical studies, support the need for further investigation into their cardioprotective potential in patients undergoing anthracycline therapy.

**Figure 1:**
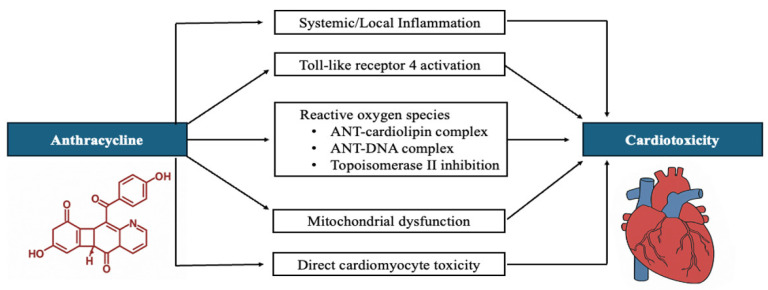
Mechanisms of Anthracycline-Induced Cardiotoxicity. This schematic illustrates the multifactorial mechanisms by which anthracyclines, a class of chemotherapeutic agents, induce cardiotoxicity. Key pathways include systemic and local inflammation, Toll-like receptor 4 activation, and generation of reactive oxygen species. These events contribute to mitochondrial dysfunction and direct cardiomyocyte toxicity, ultimately culminating in cardiac injury.

In the search for cardioprotective therapies against AIC, we theorize that GLP-1 RAs, through their pleiotropic effects, may offer cardioprotection and reduce the incidence of AIC. We explore the mechanisms underlying AIC (Figure 1), particularly inflammation and reactive oxygen species (ROS), and present evidence that GLP-1 RAs may attenuate both systemic and cardiac inflammation as well as ROS triggered by anthracycline exposure, thereby offering a potential strategy for AIC prevention.

## 2. Methods

This review was conducted as a narrative, hypothesis-generating synthesis of the existing preclinical and clinical literature. A targeted literature search was performed using major databases, including PubMed and Google Scholar, covering publications through November 2025. Search terms included but were not limited to combinations of “anthracycline-induced cardiotoxicity,” “cardio-oncology,” “glucagon-like peptide-1 receptor agonists,” “GLP-1 RA,” “inflammation,” “toll-like receptors,” and “oxidative stress.”

Studies were selected based on relevance to the mechanistic pathways of anthracycline-induced cardiac injury and the biological and clinical effects of GLP-1 RAs. Priority was given to randomized controlled trials, meta-analyses, and well-characterized preclinical models, with additional inclusion of observational and translational studies where clinical data were limited. Non-English-language publications were excluded. Conference abstracts were reviewed and cited when full manuscripts were unavailable and used as appropriate to contextualize emerging evidence, rather than to support definitive conclusions.

## 3. Anthracycline-Induced Inflammation

### 3.1. Systemic Inflammation in Anthracycline Cardiotoxicity

The mechanism of action of anthracyclines is multimodal: the major effects are to inhibit topoisomerase II, directly modify deoxyribonucleic acid (DNA), which inhibits replication and synthesis, and increase the generation of free radicals, which also disrupt normal cellular functions and increase the likelihood of apoptosis or necrosis. The effects are directly related to the frequency of cell cycling, with the greatest toxicity manifesting in cell lines with high metabolic activity or frequent replication.

Irrespective of the degree of left ventricular dysfunction, patients with clinical heart failure exhibit elevated levels of circulating pro-inflammatory cytokines (TNF-α, IL-6, IL-1) compared to individuals without heart failure [13]. Elevated C-reactive protein levels are associated with a higher risk of developing heart failure, greater disease severity, and increased mortality [14,15]. In cancer patients, underlying comorbidities in addition to iatrogenic insults, such as anthracycline exposure, contribute to the generation of ROS, which in turn promotes chronic inflammation and maladaptive left ventricular remodeling [16]. While systemic inflammation plays a major role in this pathologic process, leading to the classically described dilated cardiomyopathy and subsequent heart failure with reduced ejection fraction, increasing evidence highlights the contribution of specific innate immune mechanisms that drive and sustain myocardial injury. Among these, activation of pattern recognition receptors (PRRs), particularly toll-like receptors (TLRs), has emerged as a key mediator linking inflammation to heart failure pathogenesis.

### 3.2. Toll-like Receptor Activation in Anthracycline Cardiotoxicity

Anthracycline agents induce cardiomyocyte injury in a dose-dependent manner through oxidative stress, mitochondrial dysfunction, and DNA damage [17]. This cellular injury leads to apoptosis and necrosis, resulting in the release of damage-associated molecular patterns (DAMPs). Endogenous danger molecules—such as high-mobility group box 1, S100 proteins, mitochondrial DNA, and heat shock proteins—act as pro-inflammatory signals [18]. DAMPs bind to PRRs, including TLR4, which is significantly upregulated with cardiac injury and heart failure [19]. Its activation stimulates the release of pro-inflammatory cytokines—including IL-1, IL-6, and TNF-α—contributing to a sustained inflammatory cardiac milieu [20].

Overactivation of the innate immune system appears to be both a cause and a chronic consequence of heart failure [13]. Activation of TLR2, TLR4, and TLR5 is implicated in the TLR-initiated pro-inflammatory response in the heart [21]. Chronic inflammation—particularly that mediated by TLR4—has been linked to the development of ischemia–reperfusion injury, myocardial infarction, myocarditis, and heart failure [22]. Interestingly, TLR4 is the most abundantly expressed TLR in the heart [19] and has been implicated in AIC [23].

TLR4-mediated myocardial inflammation has been well studied in preclinical animal models. For example, in rodent models of myocardial infarction-induced congestive heart failure, elevated inflammatory cytokines and systemic inflammation were observed, along with increased TLR4 expression in the failing heart. Lentiviral inhibition of TLR4 reduced myocardial inflammation and subsequently was associated with less impairment in cardiac function following myocardial infarction [24]. Upregulation of TLR4 is observed in animal and human heart failure, contributing to heightened myocardial inflammation and tissue damage [19]. Another study in a rodent model of heart failure demonstrated that the TLR4/NADPH oxidase 4 (NOX4) pathway was significantly upregulated, leading to increased autophagy and ferroptosis. Knockdown of TLR4 or NOX4 markedly reduced cardiomyocyte death, improved left ventricular remodeling, and suppressed both autophagy and ferroptosis [25]. TLR4 activation in cardiomyocytes directly impairs cardiac function by reducing contractility and triggering an NF-κB-dependent inflammatory response. Furthermore, TLR4^−/−^ mice attenuated doxorubicin-induced cardiomyopathy, demonstrating its pathogenetic role in AIC [23]. All of this evidence implicates TLR4 as a mediator of immune signaling in the heart as well as a contributor to ventricular dysfunction following ischemic and oxidative stress [21].

## 4. GLP-1 RAs Attenuate Inflammation

### 4.1. GLP-1 RA Effects on Systemic Inflammation

GLP-1 RAs constitute a class of medications that mimic the physiological actions of endogenous GLP-1, an incretin hormone involved in glucose homeostasis. While initially marketed for glycemic control and weight loss, GLP-1 RAs have also been shown to modulate immune function and demonstrate widespread anti-inflammatory effects. In a systematic review and meta-analysis of fifty-two randomized controlled trials involving 4734 patients with T2DM, Ren et al. found that GLP-1 RAs significantly reduced systemic inflammatory markers, including CRP, TNF-α, IL-6, and IL-1, compared to placebo and other conventional anti-diabetic therapies [26]. Another large-scale meta-analysis by Bray et al., encompassing forty randomized controlled trials and nearly 6800 patients, demonstrated a similar reduction in pro-inflammatory markers, along with a decrease in malondialdehyde levels, a biomarker of oxidative stress [27]. Additionally, a study by Zobel et al. investigated the anti-inflammatory effects of liraglutide on the expression of inflammatory genes in peripheral blood mononuclear cells in patients with T2DM. The study found that liraglutide downregulated the expression of TNF-α and IL-1β, suggesting a modulatory effect on inflammatory gene expression [28].

### 4.2. GLP-1 RA Modulation of Toll-like Receptor Signaling

Preclinical animal studies have shown that TLR-induced inflammation is attenuated by GLP-1 receptor agonism. In a murine model of sepsis-induced cardiac dysfunction, Tirzepatide, a dual GLP-1 and glucose-dependent insulinotropic polypeptide receptor agonist, was shown to suppress cardiac inflammation by reducing levels of proinflammatory cytokines and inhibiting activation of the TLR4 signaling pathway, highlighting its potential to modulate innate immune mechanisms implicated in cardiac injury [29]. In another study, central GLP-1 receptor activation significantly suppressed TLR4-induced systemic inflammation, notably reducing plasma TNF-α levels and sepsis-related organ injury [30]. Liraglutide has also been shown to attenuate inflammation and fibrosis in diabetic kidney disease by downregulating the TLR4/MyD88/NF-κB signaling pathway [31].

## 5. Reactive Oxygen Species in the Pathogenesis of Anthracycline-Induced Cardiotoxicity

ROS are both a cause and a consequence of anthracycline-induced mitochondrial dysfunction. Elevated ROS levels damage mitochondrial DNA, proteins, and lipids, impairing the electron transport chain and reducing mitochondrial efficiency. This dysfunction, in turn, promotes further ROS production, creating a self-perpetuating cycle of oxidative stress and mitochondrial injury.

Two redox cycling pathways contribute to ROS generation from anthracyclines: (1) an enzymatic pathway involving NADPH-cytochrome P450 reductase within the mitochondrial respiratory chain; (2) a non-enzymatic, iron-driven pathway via the Fenton reaction. The heart, with the highest mitochondrial density of any tissue [32], is particularly vulnerable to this process. Cardiomyocyte injury occurs when the heart’s intrinsic antioxidant defenses are overwhelmed by persistent mitochondrial dysfunction. Several distinct mechanisms contribute to anthracycline-induced oxidative stress, including anthracycline–cardiolipin complex formation, topoisomerase II inhibition, anthracycline-DNA complexes, and direct cardiomyocyte toxicity.

### 5.1. Anthracycline–Cardiolipin Complex

Doxorubicin’s cardiotoxicity stems in part from its selective accumulation in mitochondria, where it exploits the electrochemical gradient to localize near cardiolipin-rich regions of the inner membrane [33]. This interaction compromises mitochondrial structure and function by impairing electron transport, particularly at complex I, resulting in stalled oxidative phosphorylation [34]. The consequent surge in ROS initiates a cascade of lipid peroxidation, with cardiolipin oxidation serving as a trigger for apoptotic signaling [35]. In the absence of cardiolipin, doxorubicin-induced oxidative stress and mitochondrial damage are attenuated [36].

### 5.2. Topoisomerase II Inhibition and DNA Complexes

Doxorubicin exerts its anticancer effects primarily by inhibiting topoisomerase II (Top2), leading to disruption of chromatin structure and interference with DNA replication. Doxorubicin is considered toxic to Top2 because it increases the formation of stabilized Top2-DNA covalent complexes, leading to persistent DNA double-strand breaks and subsequent death of both cancer cells and cardiomyocytes [37]. When doxorubicin stabilizes Top2β-DNA cleavage complexes in cardiomyocytes, there are DNA double-strand breaks as well as subsequent downregulation of genes involved in mitochondrial biogenesis and function. This disruption of mitochondrial homeostasis contributes to increased generation of ROS, promoting oxidative stress and cardiomyocyte death [38]. The combined effects of DNA damage and mitochondrial dysfunction contribute to excessive ROS accumulation, exacerbating cellular injury and promoting apoptosis.

### 5.3. Direct Cardiomyocyte Toxicity

Interesting data have been generated relating cardiomyocyte calcium handling, intracellular anthracycline concentration, and subsequent cardiomyocyte toxicity. Calcium channels are necessary to facilitate intracellular anthracycline concentration, and activators of calcium channels reduced intracellular concentrations [39]. This finding implicates calcium-dependent cell lineages and suggests that cells capable of an action potential may be preferentially exposed, in addition to rapidly reproducing cells and those with high degrees of genomic activity. This calcium dysregulation is also likely implicated in programmed cell death via calcium-dependent proteases and caspases. Anthracycline molecules have been shown in in vitro studies to be directly disruptive to Titin, the largest protein of the cardiomyocyte and anchor for the structural network of actin and myosin, via calpain-dependent proteolysis and other mechanisms [40].

## 6. GLP-1 RAs Attenuate Reactive Oxygen Species

Intramyocardial production of ROS plays a central role in the molecular pathogenesis of AIC. Increasing evidence suggests that GLP-1 RAs possess antioxidant properties that may mitigate this process and confer cardioprotection. Preclinical studies have provided mechanistic insight into this antioxidant effect. In HL-1 cardiomyocytes, liraglutide significantly reduced IL–1β-induced ROS production and suppressed the expression of NOX4, a key enzyme involved in cardiac oxidative stress. These effects were associated with improvements in mitochondrial function and reductions in lipid accumulation within cardiomyocytes [41]. Similarly, GLP-1 RAs attenuated hyperglycemia-induced apoptosis in human umbilical vein endothelial cells by inhibiting NOX4-mediated ROS generation [42,43]. This reversal of ROS production induced by hyperglycemia mitigates the downstream pathophysiologic consequences of uncontrolled glucose levels [44]. In rodent models of T2DM, GLP-1 RAs have been shown to reduce oxidative damage to the aorta and decrease myocardial fat deposition—an important contributor to inflammation and ROS production—while concurrently lowering cardiomyocyte apoptosis and overall oxidative stress [43,45].

These antioxidant effects extend to human studies. Patients with T2DM exhibit elevated markers of oxidative stress, including 3-nitrotyrosine, along with decreased circulating GLP-1 levels. These alterations have been linked to adverse cardiac remodeling and increased cardiovascular risk [46]. Notably, GLP-1 RA treatment has been associated with reduced ROS production in polymorphonuclear leukocytes, improved mitochondrial function, and decreased systemic inflammatory markers in these patients [47]. Furthermore, GLP-1 RAs mitigated oxidative damage in HL-1 cardiomyocytes exposed to palmitate-induced stress [46], and large-scale analyses have confirmed reductions in malondialdehyde—a biomarker of oxidative stress—with GLP-1 RA use [27].

Taken together, both preclinical and clinical data support the antioxidant potential of GLP-1 RAs, underscoring their relevance in cardioprotection against oxidative stress, particularly in populations at heightened cardiovascular risk, such as individuals with T2DM.

## 7. Discussion

AIC remains a major clinical challenge in the field of cardio-oncology. Despite decades of research and numerous therapeutic trials, dexrazoxane remains the only FDA-approved pharmacologic intervention with demonstrated cardioprotective efficacy. However, its restricted indications and concerns about potential interference with chemotherapy efficacy have limited its widespread use. Multiple other agents—including antioxidants, beta-blockers, and vitamins—have been tested but consistently failed to show reproducible benefit in randomized clinical trials [9]. The growing population of individuals in cancer survivorship faces a rising burden of cardiovascular complications, underscoring the need for targeted, mechanism-based approaches to cardioprotection.

This review explored the pathophysiologic mechanisms underlying AIC and evaluated the preclinical and clinical data supporting the potential use of GLP-1 RAs as cardioprotective agents. Anthracyclines damage the heart through a multifaceted cascade of injury, including the generation of ROS, mitochondrial dysfunction, and activation of innate immune pathways—particularly TLR4 signaling—culminating in direct cardiomyocyte injury and heart failure. Importantly, GLP-1 RAs lower circulating inflammatory markers that are closely implicated in the pathogenesis of heart failure.

GLP-1 RAs represent a mechanistically rational and clinically accessible strategy to mitigate anthracycline-induced cardiac injury. Their anti-inflammatory and antioxidant effects directly counter the molecular drivers of AIC, while simultaneously addressing the traditional cardiovascular risk factors that heighten patient vulnerability. As the field of cardio-oncology moves toward precision prevention, GLP-1 RAs deserve rigorous clinical investigation to determine whether their theoretical promise translates into meaningful cardioprotection in patients receiving anthracycline-based chemotherapy.

This theoretical framework is increasingly supported by emerging preclinical (Table 1) and clinical data. For example, in a rodent model, co-administration of semaglutide with doxorubicin resulted in significantly reduced histologic evidence of cardiac injury, including fewer degenerative myocardial cells and diminished infiltration of inflammatory cells [48]. Similarly, another study showed that GLP-1 treatment significantly attenuated doxorubicin-induced cardiac dysfunction, improving systolic performance and reducing myocardial fibrosis. These protective effects were linked to the modulation of macrophage polarization within cardiac tissue. Doxorubicin shifted macrophage populations toward a pro-inflammatory M1 phenotype, while GLP-1 promoted a reparative M2 phenotype by enhancing nuclear translocation of peroxisome proliferator-activated receptor-γ (PPAR-γ) and inhibiting NF-κB activation [49]. Another rodent model demonstrated that semaglutide mitigated doxorubicin-induced cardiotoxicity by improving mitochondrial function, specifically suppressing BCL2/adenovirus E1B 19 kDa protein–interacting protein 3 (BNIP3)-mediated dysfunction [50]. Pretreatment of rats with exenatide prior to doxorubicin administration attenuated left ventricular dysfunction, as demonstrated by improved echocardiographic parameters [51]. In doxorubicin-exposed rats, treatment with liraglutide reduced the elevation of cardiac injury biomarkers, including troponin and creatine kinase-MB, while also increasing levels of the antioxidant enzyme superoxide dismutase [52]. Finally, tirzepatide significantly mitigated doxorubicin-induced cardiotoxicity by activating phosphatidylinositol 3-kinase/protein kinase B (PI3K/Akt) signaling, which in turn reduced oxidative stress, inflammation, and cardiac injury [53].

Notably, the cardioprotective effects of GLP-1 RAs may depend on the anthracycline dosing regimen. Chronic doxorubicin protocols involving repeated doses over several weeks have shown that liraglutide attenuates oxidative stress, inflammation, and apoptosis [52]. In contrast, acute single-dose models of doxorubicin failed to demonstrate cardioprotection [54]. These findings suggest that the timing, duration, and intensity of anthracycline exposure may influence the efficacy of GLP-1 RAs, which could be more cardioprotective in preventing chronic rather than acute AIC.

While GLP-1 RAs demonstrate mechanistic and preclinical promise for mitigating AIC, their high cost and limited accessibility pose barriers to widespread clinical use. Future research should evaluate not only their efficacy and safety but also cost-effectiveness and strategies to improve accessibility, ensuring that promising preclinical findings can be translated into practical, real-world cardio-oncology care.

### Emerging Clinical Data

Regarding clinical data, in a recent retrospective cohort study of approximately 724 patients with breast cancer and T2DM treated with anthracyclines or HER2 inhibitors, the use of GLP-1 RAs was associated with significantly lower rates of major adverse cardiovascular events, heart failure incidence and hospitalization, and all-cause mortality [55]. In a large propensity score-matched cohort of over 5000 patients with obesity or T2DM undergoing anthracycline-based chemotherapy, GLP-1 RA use was associated with significantly reduced rates of heart failure exacerbation, hospitalizations, and all-cause mortality [56]. Similarly, in a retrospective cohort of 2282 cancer patients with cancer therapy-related cardiac dysfunction, propensity-matched analysis comparing 201 GLP-1 RA users to 201 non-users demonstrated significantly lower risks of all-cause hospitalization (HR 0.617), acute heart failure events (HR 0.612), and acute renal failure (HR 0.577) over a mean follow-up of 295 days in patients receiving GLP1 RAs, without differences in all-cause mortality [57]. More recently, in a retrospective TriNetX-based cohort of 4982 patients with cancer therapy-related cardiac dysfunction, 837 individuals who received GLP-1 RAs demonstrated significantly improved clinical outcomes compared with matched non-users. GLP-1 RA therapy, given alongside guideline-directed medical therapy, was associated with lower all-cause mortality (HR 0.57; 95% CI 0.43–0.77; *p* < 0.001), fewer acute heart failure exacerbations (HR 0.69; 95% CI 0.56–0.85; *p* < 0.001), and reduced all-cause hospitalizations (HR 0.83; 95% CI 0.72–0.96; *p* = 0.009) [58].

Although compelling, the evidence supporting GLP-1 RAs in the context of AIC is still largely preclinical or extrapolated. Clinical trials in cardio-oncology are needed to validate this therapeutic potential. Importantly, GLP-1 RAs are already FDA-approved for T2DM and weight management, with established safety profiles, making them feasible candidates for drug repurposing in this setting. In current clinical practice, GLP-1 RAs would primarily be used in patients who already meet indications for diabetes or obesity management, while their broader prophylactic use in high-risk oncology patients remains investigational. Moreover, as interest grows in leveraging cardiometabolic therapies such as SGLT2is and GLP-1 RAs for cardiovascular risk reduction in oncology, this approach may represent a paradigm shift from reactive management of chemotherapy-induced heart failure to prevention. This is especially relevant for patients with baseline cardiovascular risk factors or prolonged exposure to high-dose anthracyclines.

## 8. Limitations

It is important to acknowledge several limitations in the current review. Much of the mechanistic and preclinical data are derived from animal models, which may not fully translate to human physiology. Clinical evidence on the use of GLP-1 RAs for AIC remains limited, with most studies being retrospective. It is unclear whether GLP-1 RAs may interact with chemotherapy efficacy, and there is a need for studies to evaluate potential effects on tumor response or treatment outcomes. These agents may also exacerbate existing vulnerabilities in this population, such as promoting further weight loss, which is particularly concerning for patients already at risk for cancer-associated cachexia. Additionally, study populations have been heterogeneous with respect to cancer type, treatment regimens, and comorbidities, making it difficult to generalize findings. Variability in GLP-1 RA agents, dosing strategies, and short follow-up durations further limit conclusions. Potential drug interactions and polypharmacy in oncology patients may complicate safety and tolerability assessments. Overall, these limitations underscore the need for further prospective studies and well-designed randomized clinical trials to establish the safety, efficacy, and optimal use of GLP-1 RAs in this population.

The tolerability and safety of GLP-1 RAs in oncology patients require careful consideration, particularly in those with low body weight or cancer-related cachexia. Potential interactions with chemotherapy-induced nausea, vomiting, and gastrointestinal toxicity should be evaluated. Future studies will also need to determine the optimal timing of GLP-1 RA initiation relative to anthracycline exposure and the appropriate duration of therapy to balance cardioprotective benefits with safety.

## 9. Conclusions

Anthracyclines remain essential agents in the treatment of numerous malignancies but are limited by their dose-dependent cardiotoxic effects, which contribute significantly to long-term morbidity and mortality in cancer survivors. Despite decades of investigation, dexrazoxane remains the only FDA-approved pharmacologic agent for AIC prevention and its use is restricted to a narrow subset of patients. As such, the need for novel, mechanism-based, and widely applicable cardioprotective strategies is urgent.

GLP-1 RAs represent a promising class of therapeutics with pleiotropic cardiovascular benefits. Emerging evidence suggests that GLP-1 RAs not only modulate traditional cardiovascular risk factors but also directly counteract the molecular mechanisms underlying AIC, namely inflammation, toll-like receptor signaling, and reactive oxygen species-mediated mitochondrial injury. Preclinical models demonstrate their capacity to preserve cardiac structure and function in the setting of anthracycline exposure, while recent retrospective clinical data support their potential cardioprotective effects in patients with cancer and cardiometabolic disease.

Although the current body of evidence is compelling, it remains largely preclinical or observational. It is important to note that while preclinical models provide mechanistic insights into GLP-1 RA-mediated cardioprotection, cardiovascular outcome trials in diabetes or obesity offer indirect evidence, and data specifically in anthracycline-exposed patients remain limited and largely observational. Well-designed prospective clinical trials are essential to determine whether GLP-1 RAs can be safely and effectively repurposed for the prevention of anthracycline-induced cardiac injury. Given their established safety profile, widespread clinical use, and mechanistic rationale, GLP-1 RAs represent a promising frontier in cardio-oncology and may usher in a new era of proactive cardiovascular care for patients undergoing cancer treatment.

## Figures and Tables

**Table 1:** Preclinical Animal Studies of GLP-1 Receptor Agonists in Anthracycline-Induced Cardiotoxicity. This table summarizes key preclinical investigations assessing the cardioprotective effects of various GLP-1 receptor agonists in rodent models of anthracycline-induced cardiotoxicity. Across studies, agents including semaglutide, exenatide, liraglutide, and tirzepatide demonstrated beneficial effects through diverse mechanisms such as mitigation of mitochondrial dysfunction, enhancement of autophagy, modulation of inflammatory signaling, and activation of cardioprotective pathways. Most studies reported improved cardiac structure and function, though one study found no benefit from liraglutide pretreatment in an acute injury setting.

Author, Year	GLP-1 RA	Proposed Mechanism/Pathway	Model	Outcome Measured	Key Findings
Kyung Hye Lee, 2017 [51]	Exenatide	Enhanced autophagy	Rats	Cardiac function (Ejection fraction [EF], Fractional shortening [FS]); histology; autophagy markers (LC3-II, Beclin-1); apoptosis markers (TUNEL, caspase-3)	Preserved cardiac function; reduced cardiomyocyte damage
Noha A.T. Abbas, 2017 [52]	Liraglutide	Activation of Akt/GSK-3β signaling	Rats	Cardiac injury biomarkers (troponin I, CK-MB); apoptosis markers (caspase-3, Bcl-2); oxidative stress markers (MDA, SOD); inflammatory cytokines (TNF-α, IL-6); histopathology	Improved cardiac performance and reduced cardiomyocyte apoptosis
Xiaoping Li, 2024 [50]	Semaglutide	BNIP3-mediated mitochondrial dysfunction	Mice	Mitochondrial outcomes (membrane potential, ATP, morphology); apoptosis markers; cardiac injury markers; Cardiac function (EF, FS)	Improved mitochondrial integrity and reduced myocardial injury
Ling Chen, 2024 [53]	Tirzepatide	PI3K/Akt activation; inhibition of oxidative stress and inflammation	Mice	Inflammatory markers (TNF-α, IL-6); oxidative stress markers (ROS, MDA); histopathology	Reduced inflammation and oxidative stress with improved cardiac performance
Carolina R. Tonon, 2024 [54]	Liraglutide	No significant cardioprotection in acute injury	Rats	Cardiac structure and function (EF, FS, LV dimensions); histopathology	No improvement in myocardial structure or function
Raz Muhammed Hasmalalih, 2024 [48]	Semaglutide	Not specified (general cardioprotection)	Rats	Histopathology; Cardiac injury biomarkers (troponin, CK-MB)	Reduced histological evidence of doxorubicin-induced myocardial damage
Lujin Wu, 2025 [49]	GLP-1 (unspecified)	Modulation of macrophage polarization (M1 → M2)	Mice	Immune outcomes (M1/M2 macrophage markers); inflammatory cytokines (IL-1β, TNF-α); cardiac injury markers; Cardiac function (EF, FS)	Reduced inflammatory response and improved post-injury remodeling

## Data Availability

No new data were created or analyzed in this study. Data sharing is not applicable to this article.

## Abbreviations

The following abbreviations are used in this manuscript:

AIC	anthracycline-induced cardiotoxicity
BNIP3	BCL2/adenovirus E1B 19 kDa protein–interacting protein 3
DAMPS	damage-associated molecular patterns
DNA	deoxyribonucleic acid
FDA	Food and Drug Administration
GLP-1 RAs	glucagon-like peptide-1 receptor agonists
NOX4	NADPH oxidase 4
PPAR-γ	peroxisome proliferator-activated receptor-γ
PI3K/Akt	phosphatidylinositol 3-kinase/protein kinase B
ROS	reactive oxygen species
SGLT2is	sodium–glucose cotransporter 2 inhibitors
T2DM	type II diabetes mellitus
TLRs	toll-like receptors
Top2	topoisomerase II
